# Cytocompatibility of Modified Silk Fibroin with Glycidyl Methacrylate for Tissue Engineering and Biomedical Applications

**DOI:** 10.3390/biom11010035

**Published:** 2020-12-29

**Authors:** Heesun Hong, Ok Joo Lee, Young Jin Lee, Ji Seung Lee, Olatunji Ajiteru, Hanna Lee, Ye Ji Suh, Md Tipu Sultan, Soon Hee Kim, Chan Hum Park

**Affiliations:** 1 Nano-Bio Regenrative Medical Institute, College of Medicine, Hallym University, Chuncheon 24252, Korea; heesun181025@gmail.com (H.H.); vudckd@hanmail.net (O.J.L.); skws789@naver.com (Y.J.L.); dbrghrl@naver.com (J.S.L.); ajiteruolatunji@gmail.com (O.A.); dlgkssk1995@naver.com (H.L.); olivia1995@naver.com (Y.J.S.); tipubge@yahoo.com (M.T.S.); soonheekkim@gmail.com (S.H.K.); 2 Departments of Otorhinolaryngology-Head and Neck Surgery, Chuncheon Sacred Heart Hospital, School of Medicine, Hallym University, Chuncheon 24253, Korea

**Keywords:** silk fibroin, chemical modification, glycidyl methacrylate, dialysis periods, cytocompatiblity, tissue engineering

## Abstract

Hydrogel with chemical modification has been used for 3D printing in the biomedical field of cell and tissue-based regeneration because it provides a good cellular microenvironment and mechanical supportive ability. As a scaffold and a matrix, hydrogel itself has to be modified chemically and physically to form a β-sheet crosslinking structure for the strength of the biomaterials. These chemical modifications could affect the biological damage done to encapsulated cells or surrounding tissues due to unreacted chemical residues. Biological assessment, including assessment of the cytocompatibility of hydrogel in clinical trials, must involve testing with cytotoxicity, irritation, and sensitization. Here, we modified silk fibroin and glycidyl methacrylate (Silk-GMA) and evaluated the physical characterizations, residual chemical detection, and the biological effect of residual GMA depending on dialysis periods. Silk-GMA depending on each dialysis period had a typical β-sheet structure in the characterization analysis and residual GMA decreased from dialysis day 1. Moreover, cell proliferation and viability rate gradually increased; additionally, necrotic and apoptotic cells decreased from dialysis day 2. These results indicate that the dialysis periods during chemical modification of natural polymer are important for removing unreacted chemical residues and for the potential application of the manufacturing standardization for chemically modified hydrogel for the clinical transplantation for tissue engineering and biomedical applications.

## 1. Introduction

Hydrogels are a bio ink with a synthesizing 3D crosslinked hydrophilic structure with a 3D scaffold for bioprinting. They have been used as a cell matrix and provide a supportive biological microenvironment along with mechanically modified material that was originally native to the tissue [1,2,3]. There are many reported natural and synthetic biomaterials, including fibrinogen, agarose, gelatin, hyaluronic acid, alginate, and silk. Among these, silk is an excellent biomaterial for various biomedical applications, including bone [4] and cartilage regenerations [5], artificial blood vessel [6,7], skin [8] and wound dressing material [9], controlled drug delivery scaffold [10], and nano-particles [11]. 

Silk fibroin (SF) isolated from *Bombyx mori* silkworm cocoons has been used as a scaffold material in various studies for tissue engineering and biomedical applications due to its biocompatibility, excellent mechanical properties, biodegradability, and simple aqueous process ability [12,13]. The fabrication techniques of silk-based biomaterials plays a critical role in its biocompatibilities. The degumming process involves sericin removal from the silk fibers with a strong ionic aqueous solution. This process is important for assuring the biocompatibility and the cytocompatibility of the biomaterials [14]. Among the strong ionic compounds, sodium carbonate (Na_2_CO_3_) is used for processing into various biomaterial formats, such as scaffolds, films, hydrogels, and nanoparticles [15]. 

The SF structure consists of the amino acid sequences, Gly-Ser-Gly-Ala-Gly-Ala [15]. These amino acid sequences can control the crystallinity with the stability of the β-sheets and improve the mechanical properties of SF. The β-sheet structure of SF can be controlled by enzymatic, physical, and chemical cross-linking methods, such as glycidyl methacrylate (GMA), glutaldehyde, and methacrylate (MA), leading to materials with controlled crystallinity and mechanical strength concerning SF [11,12,13]. Among these SF can be processed into various structures of amino-acid modification processes with methacrylate groups and amine residue groups of hydrogels including gel, membrane, and porous sponges with aqueous processing and light-polymerization with a photoinitiator, such as LAP (Lithium phenyl(2,4,6-trimethylbenzoyl) phosphinate) with a 3D printing application [16,17]. In particular, SF with GMA can be modified with functional groups, such as methacryloyl, for further modification with photo-crosslinking under 3D printing. Methacyloyl modification occurs with the amine, hydroxyl, and carboxyl groups in SF with specific reaction conditions such as pH. GMA modification is primarily carried out with an epoxide ring opening to enhance the methacryloyl modification, and also to react with the primary amines of lysine residues. The degree of methacryloyl substitution is associated with the concentration of GMA. The methacryloyl substitution in the hydroxyl and carboxyl groups may increase the number of cross-linkable sites and thus the photo-crosslinking density of silk fibroin. These degrees of methacrylation by GMA reaction, concentration of photoinitiator, and photo-crosslinking time affect the mechanical properties of hydrogel as a biomaterial [16].

Chemically modified SF hydrogel could affect the damage done to the encapsulated cells or the surrounding tissues after implantation due to the unreacted chemical residues and released chemical debris during the chemical modifications with the cross-linker. The assessment of biocompatibility and cytocompatibility could issue a challenge for the application to biomedical and tissue engineering as a medical device. Assessment of the biocompatibility for clinical application has limitations. The relevant limitations include factors like the chemical–physical characteristics of the biomaterials, the contact tissue type, and the duration of contact. An appropriate guidance or risk-management could be provided for choosing biomaterials and relevant biocompatibility test methods [18,19].

ISO 10993-5, Part 5; tests for in vitro cytotoxicity in biological evaluation of medical devices (International Organization for Standardization, third edition, 2009) suggest several assessment criteria: (i) Assessment of cell damage by morphological means, (ii) measurements of cell damage, (iii) measurements of cell growth, and (iv) measurements of specific aspects of cellular metabolism. These were specified with the following methods: (i) The incubation of cultured cells with a device, and (ii) extracts of a device through diffusion. These specified methods are for determining the biological response of encapsulated cells in biomaterials using appropriate biological parameters with in vitro conditions [20]. 

Biological assessment, including assessment of the biocompatibility and cytocompatibility of hydrogel for humans in clinical trials, must involve testing with cytotoxicity, irritation, and sensitization. The appropriate dialysis period concerning the modified SF with chemicals is important for removing the unreacted chemical cross-linker or aqueous chemicals, such as lithium bromide (LiBr), for the biosafety and biocompatibility of hydrogel as a biomaterial. The experiments and analysis of the cytocompatibility of the modified SF with chemicals has not been reported before. We hypothesized that residual GMA from modified SF with GMA (Silk-GMA) could be harmful to the encapsulated cells or the surrounding tissues after implantation in in vivo circumstances. We investigated the physical characterizations, biological effects, including cell proliferation, and necrotic/apoptotic effects of residual GMA depending on dialysis periods after reaction with SF and GMA for the application with 3D bioprinting technology related to tissue engineering and biomedical applications.

## 2. Materials and Methods 

### 2.1. Materials 

Glycidyl methacrylate (GMA, CAS No. 106-91-2, MW 142.1546 g/mol), ethyl acetate solvent (EtOAc, CH_3_COOC_2_H_5_, purity ≥ 99.9%, CAS No. 141-78-6), and deuterium oxide (D_2_O, 99.9 atom%D) were purchased from Sigma-Aldrich (St. Louise, MO, USA). *Bombyx mori* cocoons were collected from the Rural Development Administration, Korea. Sodium carbonate (Na_2_CO_3_, Cas No. 497-19-8, MW 105.9888 g/mol) was purchased from Daejung (Seoul, Korea), and lithium bromide (LiBr, Cas No. 24120-01, MW 86.845 g/mol) from Kanto chemical Co., Inc. (Tokyo, Japan). The miracloth was purchased from Millipore (Burlington, MA, USA), and the dialysis membranes with molecular weight cutoff (MWCO) size of 12–14 kDa were from Spectrumlabs (Rancho Dominguez, CA, USA). The tubes for NMR acquisition were from Norell (Morganton, NC, USA). Dulbecco’s modified Eagle medium-high glucose (DMEM-high glucose) culture media was purchased from Welgene (Seoul, Korea), and fetal bovine serum (FBS) from Corning (Glendale, AZ, USA). The antibiotic/antimycotic (AA) solution, TrypLE^TM^ express as a cell-dissociation enzyme, and 0.4% trypan blue solution were obtained from GIBCO (Grand Island, NY, USA). Live & Dead assay kit and FITC Annexin V/Dead cell apoptosis kit were purchased from Invitrogen (Waltham, MA, USA), and the Cell Counting Kit-8 assay (CCK-8) was obtained from EnzoBiochem (New York, NY, USA).

### 2.2. Silk-GMA Preparations

**Figure 1:**
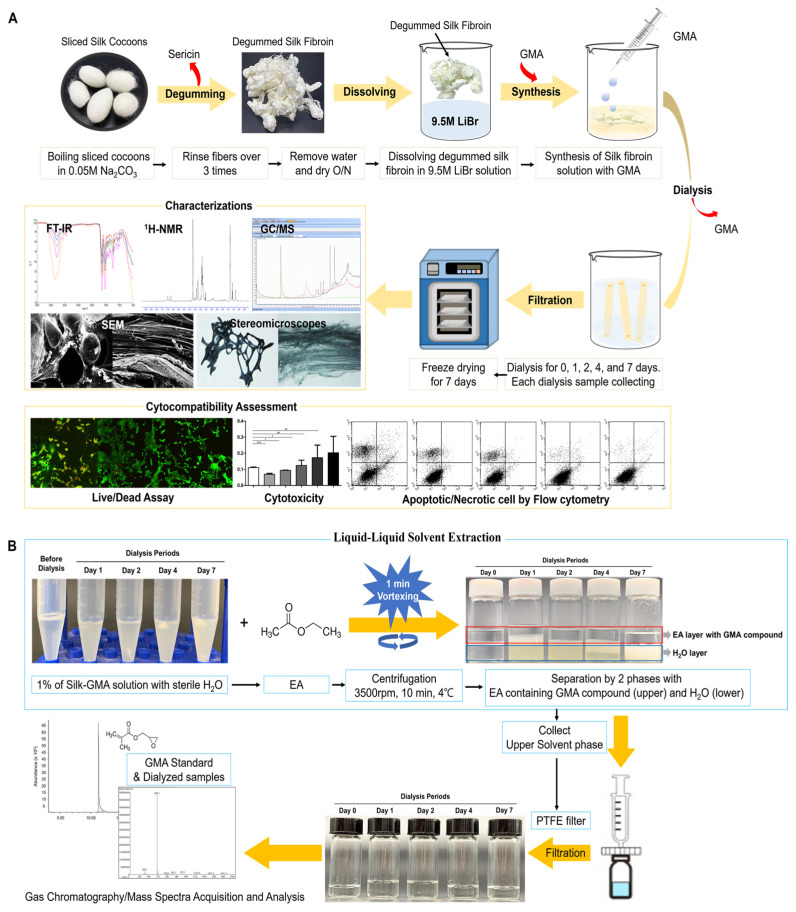
A schematic illustration of the biocompatibility screening procedures for modified silk fibroin with glycidyl methacrylate (Silk-GMA). (**A**). For the manufacturing, SF (acronym definitions below) was chemically modified with GMA. Degummed silk was dissolved in 9.3 M LiBr and the solutions were set to react with GMA for 6 h at 60 °C. Then, it was dialyzed with distilled water at room temperature for seven days and then freeze-dried for up to seven days. (**B**). The liquid–liquid extraction method was used for application to GC-MS to detect the residual glycidyl methacrylate on each dialysis modified SF with GMA samples. Using the 2-phase separation method with EA, the upper phase was collected for GC-MS acquisition. On GC-MS acquisition, each extracted sample was injected and helium as a carrier gas and capillary column was used. The scanning range was between 35 and 200 *m*/*z* (Table 1). SF, silk fibroin; GMA, glycidyl methacrylate; Silk-GMA, modified silk fibroin with glycidyl methacrylate; LiBr, lithium bromide; EA, ethyl acetate; GC-MS, gas chromatography/mass spectra; PTFE, polytetrafluoroethylene.

Figure 1A presents a schematic summary of the modified silk fibroin with glycidyl methacrylate (Silk-GMA), according to our previous reports [5,16].

#### 2.2.1. Silk Fibroin Extraction

To make degummed silk fibroin (SF) by removing the exterior sericin protein, 40 g of sliced *Bombyx mori* cocoons (Jeonju, Jeonrabukdo, Korea), in four pieces, were boiled in 1 L of 0.05 M sodium carbonate (Na_2_CO_3_, Daejung, Seoul, Korea) solution at 100 °C for 1 h, and washed with distilled water at least three times. Then, degummed SF was dried in a dry oven for 24 h, and stored at room temperature for further experiment. 

#### 2.2.2. Preparation of Modified Silk Fibroin with Glycidyl Methacrylate (Silk-GMA)

For the synthesis of SF and GMA, 40 g of degummed SF was dissolved in 9.3 M of lithium bromide (LiBr, Kanto Chemical Co., Ltd., Tokyo, Japan) solution at 60 °C for 1 h, and 6 mL of glycidyl methacrylate (GMA, Sigma-Aldrich, St. Louise, MO, USA) was mixed with stirring at 1000 rpm for 6 h at 60 °C to cause reactions between SF and GMA. Subsequently, the solution was filtered through a miracloth (Millipore, Burlington, MA, USA) and the dialysis procedure was used with dialysis membranes (MWCO 12–14 kDa, Spectrumlabs, Rancho Dominguez, CA, USA) for dialysis periods of 1, 2, 4, and 7 days, changing the distilled water three times a day.

#### 2.2.3. Dialysis Process of Silk-GMA

The before and after for each dialysis Silk-GMA solution samples for 1, 2, 4, and 7 days were filtered through a miracloth (Millipore, Burlington, MA, USA). The collected Silk-GMA solution samples were frozen at −80 °C for at least 24 h and freeze-dried for 7 days. Freeze-dried dialyzed Silk-GMA materials were stored at 4 °C for further analysis.

### 2.3. Characterizations 

#### 2.3.1. ^1^H Nuclear Magnetic Resonance (NMR) Spectra of Silk-GMA on Various Dialysis Periods

The sample preparations for ^1^H-NMR analysis was prepared by 1 mg of various dialyzed freeze-dried Silk-GMA in 1 mL of deuterium oxide (D_2_O, Sigma-Aldrich, St. Louise, MO, USA), after that, the solution was filled in an NMR tube (Standard series, NORELL Inc., Morganton, NC, USA). ^1^H-NMR measurements were conducted using the Bruker Fourier Transform-NMR Spectrometer at a standard frequency 600 MHz with a magnetic field of 14.1 Tesla (Bruker, Billerica, MA, USA).

#### 2.3.2. Fourier Transform-Infrared Spectroscopy (FT-IR) Analysis 

Fourier Transform-Infrared Spectroscopy (FT-IR, frontier FT-IR Spectrometer, PerkinElmer, Waltham, MA, USA) analysis was performed for each dialysis freeze-dried Silk-GMA solution. Each dialysis period sample was placed directly onto the stage of the FT-IR spectrometer instrument, and each spectrum was acquired in absorbance with 32 scans, at 4 cm^−1^ resolution, and 4000 ~ 500 cm^−1^ spectral range.

#### 2.3.3. Stereomicroscopic Observations 

To detect the microstructure with spatial scales, SMZ25 (Nikon, Tokyo, Japan) was used. Briefly, the microstructure of each dialyzed freeze-dried Silk-GMA sample was captured using a glass stage plate with light from the left and right side and beneath the glass stage, and its magnified range was from 10× to 40×. 

#### 2.3.4. Scanning Electron Microscopy (SEM)

The cross-sectional microscopic observations of Silk-GMA samples of each dialysis period were performed by scanning electron microscopy (SEM, JSM-6010LV, Jeol, Tokyo, Japan). Freeze dried Silk-GMA sponge samples were fixed using a carbon conductive adhesive tape on aluminum stubs and covered with gold palladium using a sputter coat at 7 mA for 60 s. 

### 2.4. Gas Chromatography–Mass Spectrometer

#### 2.4.1. Preparation of Liquid–Liquid Solvent Extracts of Silk-GMA Hydrogel 

In 1 mL of sterilized distilled water, 0.1 g of each dialysis of modified Silk-GMA samples was dissolved, and then mixed with the same volume of ethyl acetate (EtOAc, CH_3_COOC_2_H_5_, purity ≥ 99.9%, Sigma-Aldrich, St. Louise, MO, USA). Subsequently, each mixture of the Silk-GMA solution and EtOAc was filtered through a syringe filter with the hydrophobic polytetrafluoroethylene membrane (PTFE, 0.45 µm, Millipore-Sigma, St. Louis, MO, USA). The filtered extracts were used for gas chromatographic–mass spectrometric acquisition. As a standard, glycidyl methacrylate (GMA, Sigma Aldrich, St. Louise, MO, USA) was used with the original solution and denatured GMA with a high temperature of 60 °C. For the denaturation, 100 µL of GMA was kept at 60 °C for 6 h for the same conditions of reaction with silk fibroin and glycidyl methacrylate. After the reaction, the denatured GMA was kept in the refrigerator for the acquisition of gas chromatographic–mass spectrometry (Figure 1B).

#### 2.4.2. Instrument and Analytical Condition 

Gas chromatographic–mass spectrometric acquisition was performed using Agilent Technologies 7820 A Gas Chromatograph with a 5977E MSD Mass Spectra mass selective detector (Agilent Technologies Inc., Santa Clara, CA, USA) in full scan mode, which has a scanning range between 35 and 200 mass to charge ratio (*m/z*). The capillary column used for separation was the Fused silica HP-5ms GC column (Agilent Technologies Inc., Santa Clara, CA, USA) with a length of 30 m, a membrane thickness of 0.25 µm, and an inner diameter (ID) of 0.25 mm. The injection temperature was set at 280 °C, and subsequently injected with 1 µL of each extraction from dialyzed samples. The split ratio was 10:1. Oven temperature during the analysis was set at 60 °C for 5 min, increased up to 200 °C for 5 min at a rate of 5 °C per minute, and maintained at 230 °C throughout the experiments. Helium (purity ≥ 99.99%) was used as a carrier gas with a flow rate of 1.0 mL/min. The ChemStation F.01.00.1903 and MassHunter GC/MS Acquisition B.07.00 SP2. 1654 software (Agilent Technologies, Inc., Santa Clara, CA, USA) were used for the analysis of GC and MS results, respectively. Regarding the data acquisition conditions for the mass spectra of the mass selective detector, the detector was turned off from injection for 3.5 min to protect the detector from a huge amount of solvent eluted; then the detector was turned on. An *m*/*z* (mass/charge) appropriate specifically for the detection objective were only acquired at each acquiring step after 3.5 min of injection (Table 1). 

### 2.5. Biological Analysis

#### 2.5.1. Cell Line

The NIH 3T3 mouse embryonic fibroblast cell line was obtained from ATCC (USA). NIH 3T3 cells were cultured for three days in a 100 mm culture dish (SPL, Pocheon-si, Gyeonggi-do, Korea) containing Dulbecco’s modified Eagle medium-high glucose (DMEM-high glucose, Welgene, Seoul, Korea) supplemented with 10% heat inactivated fetal bovine serum (FBS, Corning, Glendale, AZ, USA) and 1% antibiotic/antimycotic (AA, GIBCO, Grand Island, NY, USA) solution. NIH 3T3 cells were maintained in a 5% CO_2_ supplied incubator at 37 °C with the medium changing every 3–4 days. Cells were detached using TrypLE^TM^ express (GIBCO, Grand Island, NY, USA), then counted with 0.4% trypan blue (GIBCO, Grand Island, NY, USA) exclusion by LUNA II automatic cell counter (Logos, Seoul, Korea). 

#### 2.5.2. Preparation of Liquid Extracts of Each Dialyzed Silk-GMA Solution 

The biosafety test for the liquid extract of dialyzed Silk-GMA samples was prepared so that 1 g of Silk-GMA sponge of each dialysis period was mixed with 10 mL of 10% FBS containing a DMEM-high glucose culture medium, and filtered with a syringe filter (0.20 um, GVS., Sanford, ME, USA). Each filtered Silk-GMA solution was used for further experiments concerning biological assessment. 

#### 2.5.3. Cell Proliferation Assay

A Cell Counting Kit-8 assay (CCK-8, EnzoBiochem, New York, NY, USA) was carried out according to the manufacturer’s instructions. In brief, NIH 3T3 cells were seeded at a density of 2 × 10^3^ cells per well of a 96 well plate with a DMEM-high glucose supplemented 10% FBS and a 1% AA solution, and incubated for three days at 37 °C in an incubator supplied with 5% CO_2_. After cultivation, the culture medium was removed and then each well was washed with Dulbecco’s phosphate buffered saline (D-PBS, Corning, Glendale, AZ, USA) three times; 100 μL of each extract (0.1 g/mL) from various dialyzed Silk-GMA was added per well, and incubated for 72 h with each dialyzed Silk-GMA extract. After 72 h of cultivation, each extract was removed, and 10 μL of CCK-8 assay reagent was added per well. After incubation for 3 h at 37 °C in a CO_2_ incubator, the absorbance was measured at 450 nm using an ELISA microplate reader (BioTek, Winooski, VT, USA).

#### 2.5.4. Cell Viability 

The Live & Dead assay kit (Invitrogen, Waltham, MA, USA) was used for the examination of cell viability. In brief, after incubation for 72 h of each dialyzed Silk-GMA solution (0.1 g/mL) and NIH 3T3 cells at 1 × 10^4^ cells per well of a 24 well plate, all of each dialyzed Silk-GMA solution aspirated and the attached cells were washed with D-PBS (Corning, Glendale, AZ, USA). Then they were incubated with D-PBS containing 2 μM Calcein-AM and 4 μM ethidium homodimer-1 for 30 min in a 5% CO_2_ incubator conditioned at 37 °C. After incubation, stained cells that had reacted with each dialyzed Silk-GMA solution were visualized and imaged using a fluorescent microscope (Eclipse 80i, Nikon, Tokyo, Japan). 

#### 2.5.5. Flow Cytometric Analysis for Detection of Necrotic and Apoptotic Population

The necrotic and apoptotic cell population was evaluated using a dead cell apoptosis kit with Annexin V conjugated with fluorescein isothiocyanate (FITC) and propidium iodide (PI) (Annexin V/Dead cell Apoptosis Kit, Molecular Probes, Eugene, OR, USA) by flow cytometry. In brief, the NIH 3T3 cell line was cultured in a monolayer, and 10 mL of each prepared dialyzed Silk-GMA solution (0.1 g/mL) was added onto monolayered cells and then cultured for 72 h in a 5% CO_2_ supplied incubator at 37 °C. After 72 h of cultivation, each dialyzed Silk-GMA solution was removed and washed twice with D-PBS, and then cultured cells were trypsinized with TrypLE^TM^ express (GIBCO, Grand Island, NY, USA) as a cell dissociation reagent, then counted with 0.4% trypan blue (GIBCO, Grand Island, NY, USA) using a LUNA II automatic cell counter (Logos, Seoul, Korea); 1 × 10^6^ cells/mL of NIH 3T3 cells were washed in cold 1X phosphate buffered saline (PBS, Sigma Aldrich, St. Louise, MO, USA) and resuspended in PBS. Each aliquot of 100 μL was transferred to a 5 mL tube (Corning, Glendale, AZ, USA); 5 μL of Annexin V-FITC and 5 μL of PI were added, gently vortexed, and incubated for 30 min at 4 °C in the dark. After that, 1X PBS was added to each tube and washed two times with centrifugation at 1000 rpm for 5 min. Each sample was examined using flow cytometry (FACSCalibur, BD Biosciences, San Jose, CA, USA) and analyzed with CellQuest program (BD Biosciences, San Jose, CA, USA). In brief, about 10,000 events were accumulated per sample, and quadrant settings were based on control samples with fluorescent intensity. For the gating strategies, to identify early apoptotic cells from death cells resulting from late apoptosis or necrosis, the vital dye propidium iodine (PI) was used. In this way, cells that are viable are both Annexin V−FITC and PI negative; cells that are in early apoptosis are Annexin−FITC positive and PI negative, and cells that are in late apoptosis or already dead are both Annexin−FITC and PI positive. 

### 2.6. Statistics

All experiments were repeated at least three times with each sample. GraphPad Prism 6 software (San Diego, CA, USA) was used for statistical analysis and graphical representation of the data. Student’s t-tests were performed to analyze the data. The values in this study were expressed as mean ± Standard Deviation (S.D.) from three experiments.

## 3. Results and Discussion

### 3.1. Characterization of before Dialysis and Depending on Dialysis Period of Silk-GMA 

#### 3.1.1. ^1^H-NMR Spectra 

**Figure 2:**
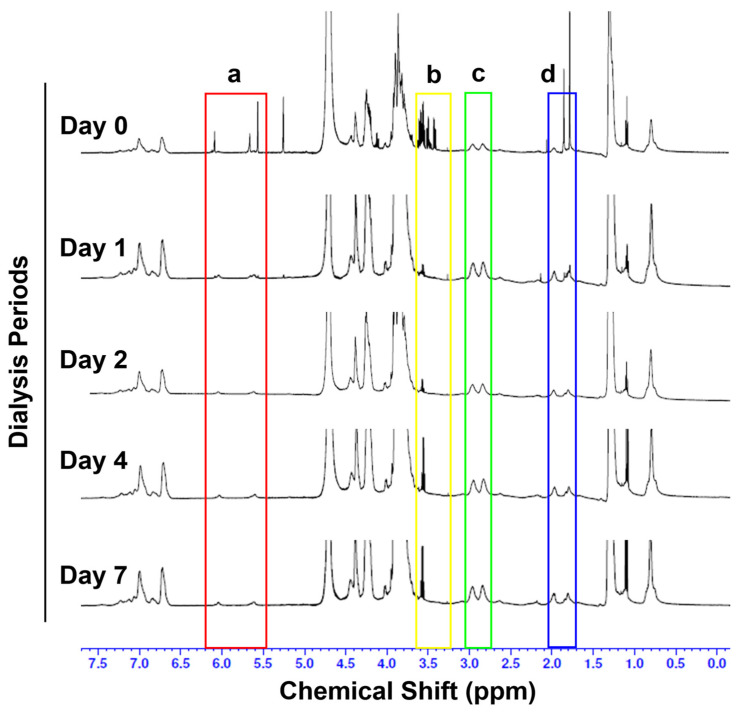
^1^H-NMR spectra of each dialysis sample from modified silk fibroin with glycidyl methacrylate. The modifications of lysine residues were demonstrated for the following: a. Methacrylate vinyl signal at δ = 6.2–5.6 ppm (red box); b. amine (δ = 3.2–3.6 ppm, yellow box); c. lysine group signal at δ = 2.9 ppm (green box); and d. methyl group at δ = 1.8 ppm (blue box) in each dialysis sample from Silk-GMA.

The differences of the chemical shift between dialysis periods of modified Silk-GMA were demonstrated using 1H Nuclear Magnetic Resonance (NMR) spectra with detection for ring opening of epoxy at GMA and nucleophilic reactions by primary amine residue on a lysine group depending on dialysis periods (Figure 2). We identified that the characteristic resonance of the methacrylate vinyl group (*δ* = 6.2−5.6 ppm) and the methyl group of GMA at *δ* = 1.8 ppm decreased following dialysis periods. In addition, the lysine signal which modified lysine groups in SF was found at the lysine signal (*δ* = 2.9 ppm) on the following dialysis periods. Dialysis day 0 showed the complex and mixed uncharacterized signal; however, each dialysis period on days 2, 4, and 7 showed that the integration of each signal was identified with measurable signals [16]. 

#### 3.1.2. Fourier-Transform Infrared Spectroscopic (FT-IR) Analysis

Fourier-transform infrared spectroscopy (FT-IR) is very sensitive to conformation modifications for silk fibroin. Each SF crystalline form shows a specific absorption band in distinct vibrational regions associated with the amide groups in proteins. The most important infrared bands for analyzing silk proteins are the amide bands: Amide I on peaks around 1620 cm^−1^ (C=O stretching), amide II on peaks around 1513 cm^−1^ (N-H bending), peaks around 1230 and 1444 cm^−1^ for amide III (C−N stretching), peaks around 3300 cm^−1^ for Amide A (hydrogen bonding), and 694 cm^−1^ for amide V. The amino acid conformation of *B. mori* silk fibroin is characterized by the peaks of β−sheet absorption around 1630, 1530, and 1240 cm^−1^, random coil conformation absorption peaks at 1650 or 1645, 1550, and 1230 cm^−1^, and an α-helix absorption peak around 1655 cm^−1^. The peaks around 3300 cm^−1^ appear due to a fluctuation in response to hydrogen bonds. Therefore, the conformation of β−sheet with related amide groups is not affected [21]. 

**Figure 3:**
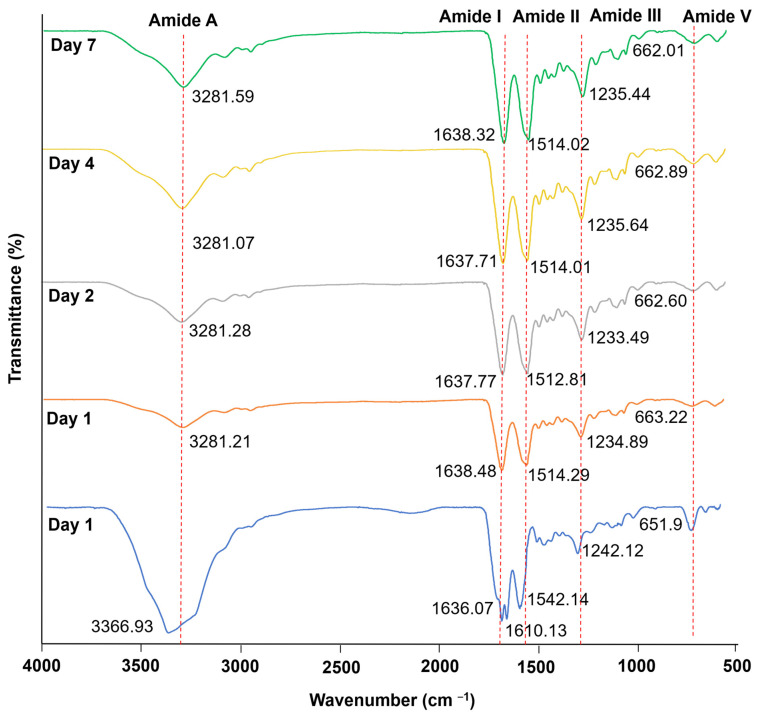
FT-IR Spectroscopic analysis of modified silk fibroin with glycidyl methacrylate on various dialysis periods. The FTIR spectra shows the presence of Amide I, II, III, and V bending at 1638 cm^−1^, 1514 cm^−1^, 1234 cm^−1^, and 662 cm^−1^ peaks, respectively, in a β−sheet structure. N-H stretching of amide A groups (about 3300 cm^−1^) overlapped with O−H absorption. Blue line, dialysis day 0; orange line, dialysis day 1; grey line, dialysis day 2; yellow line, dialysis day 4; and green line, dialysis day 7. FT-IR, Fourier transform Infrared.

The specific band in the regions related to amino acid between each dialysis sample was confirmed (Figure 3). In the results of FT−IR spectra, all samples from each dialyzed Silk-GMA have amide I at 1610 and 1638 cm^−1^, contributing to C=O stretching; amide II at 1514 cm^−1^, contributing to N-H bending; amide III at 1234 cm^−1^, contributing to C−N stretching; and amide V at 662 cm^−1^, shown in the β−sheet structure of dialysis samples from Silk-GMA, as well as the amide A group at 3300 cm^−1^. On dialysis day 0, the amide A group with the N-H stretching vibration of amide groups around 3300 cm^−1^ overlapped with the O-H stretching of hydroxyl amino acid residues. These results indicated that the hydrogen bonds of intra− or inter−molecules of amino acids affect the intensity of peaks when modified with a chemical crosslinker [21,22]. Moreover, the two distinct peaks were observed at 1610 and 1638 cm^−1^ on dialysis day 0, which corresponds to the confirmation of the α-helix and β−sheet structure. After crosslinking, the C=O band was slightly shifted to a lower wavenumber by the loss of the carboxyl group conjugation [23]. FT-IR analysis showed that a slight shift band for each amide I and II peak on dialysis day 0 was unclear; however, each sample from dialyses 1, 2, 4, and 7 on the FT−IR results has each amide group distinctly. These results indicated the β−sheet structure without conformational changes compared to the dialysis day 0 of Silk-GMA. 

#### 3.1.3. Stereomicroscopic and Scanning Electron Microscopic Findings of Freeze-Dried Silk-GMA Sponges 

**Figure 4:**
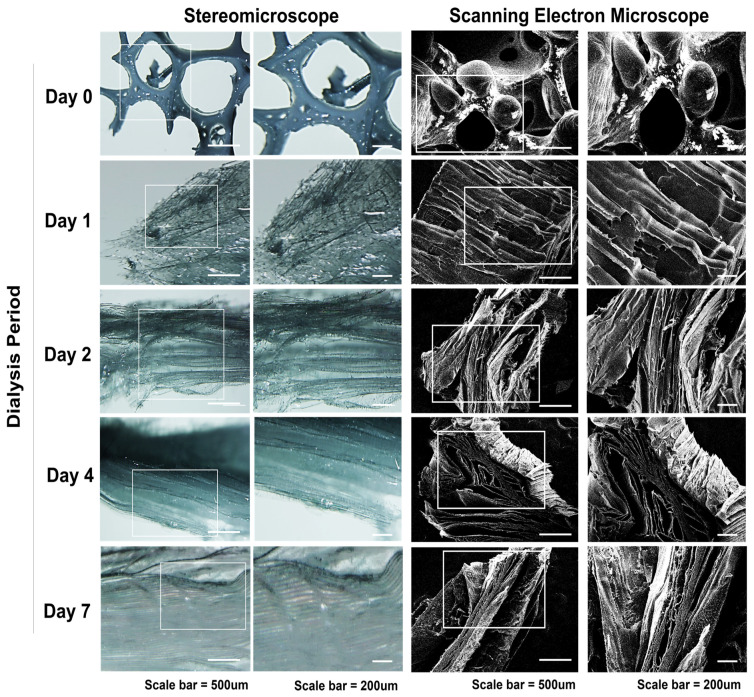
Stereomicroscopic and scanning electron microscopic (SEM) findings. Freeze-dried Silk-GMA samples from each dialysis period were demonstrated for the analysis with stereomicroscope and SEM. The uneven structures were shown on dialysis day 0; however, interconnected regular sheeted-shape microstructures were detected increasingly following dialysis periods on days 1, 2, 4, and 7. Scale bar showed 500 µM and 200 µM, separately.

The cross-sectional morphologies of each dialysis period of freeze-dried Silk-GMA samples were observed by stereomicroscope and scanning electron microscope. Dialysis day 0 showed the incomplete interconnection inside of freeze-dried Silk-GMA. This result indicated that before dialysis, Silk-GMA samples were fully saturated due to high ionic strength that controlled solvation of Silk-GMA. However, in accordance with to each dialysis period from days 1 to 7, freeze-dried Silk-GMA sponges have a highly interconnected and uniformed microstructure with a regular sheeted-shape, compared to day 0. The stereomicroscopic finding showed that dialysis day 0 has an uneven form incompletely. However, dialysis days 1 to 7 revealed the microfiber shape, and the density of the microfiber increased until dialysis day 7. At the end of the dialysis periods, the microfiber shape showed very clearly and distributed on the Silk-GMA sponges with regular distance between each microfiber. The observation of SEM showed that interconnected microstructures with regular sheeted-shape were detected (Figure 4). These results demonstrated that dialysis periods affect the formation with microstructures and interconnection of these structures with sheeted-shape through the elimination of strong salt and unreacted chemicals [24,25,26]. 

### 3.2. Gas Chromatographic–Mass Spectra Analysis 

**Figure 5:**
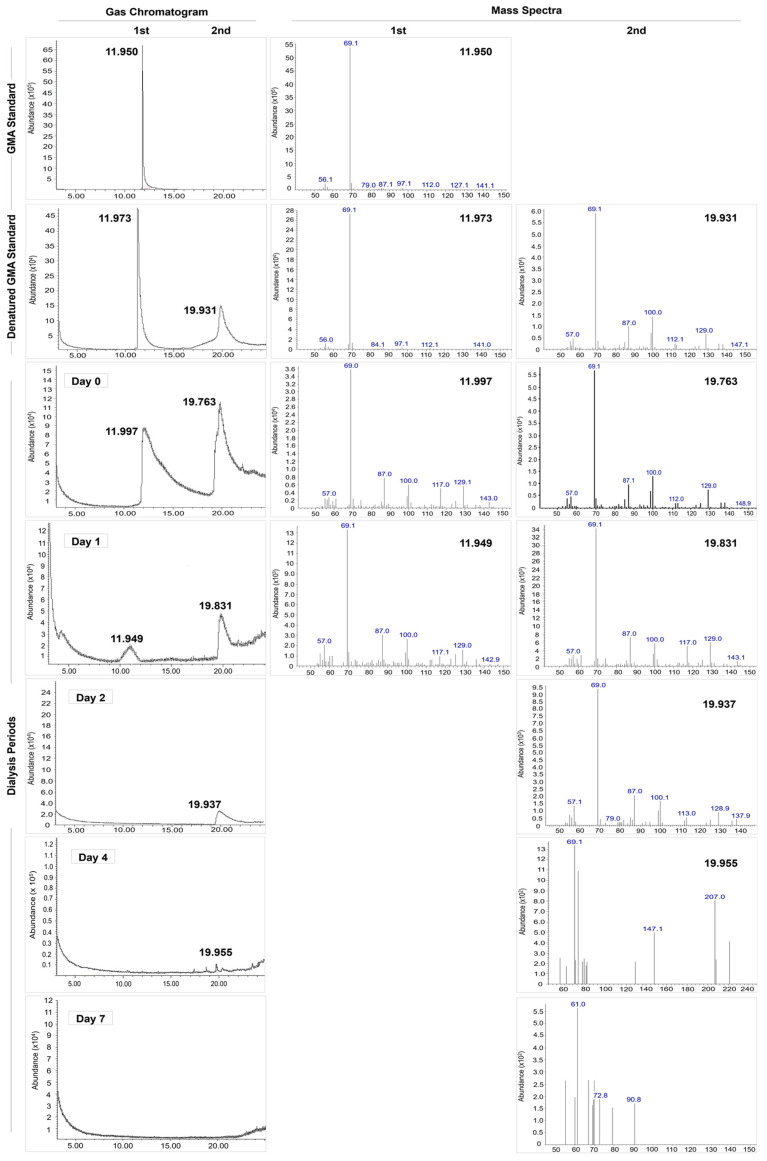
Gas Chromatography–Mass Spectra (GC-MS) analysis of residual GMA in the modified SF with GMA following dialysis periods. GMA and denatured GMA were used for a standard and denatured standard solution at 60 °C for 6 h with the same condition. The liquid–liquid solvent extraction with ethyl acetate was used for extraction of residual GMA. Each dialyzed sample from the GC-MS analysis showed that residual GMA reduced depending on dialysis periods in modified SF with GMA samples.

Gas Chromatographic–Mass Spectra (GC-MS) was performed to detect residual GMA in the Silk-GMA following dialysis periods (Figure 5). GMA and denatured GMA were used for a standard and denatured standard. Denatured GMA was made with incubation at 60 °C for 6 h. This procedure is the same denatured condition as the modification of SF with GMA (Figure 1). 

For the acquisition of the GC-MS chromatogram, the liquid–liquid solvent extraction with ethyl acetate was used for extraction of residual GMA in each dialyzed Silk-GMA. GC-MS chromatograms were shown in Figure 5 and Table 2. The standard GMA was detected with the highest intensity, and the retention time was 11.950 min. The denatured GMA results revealed an extra peak of a later retention time. These two different type of peaks were presented at 11.973 and 19.931 min of retention time. Dialysis days 0 and 1 presented the original peak at a retention time of about 11.9 min, and at the same time, another extra peak appeared at a different retention time of about 19.97 min. However, the findings of dialysis days 2 to 4 did not show original peaks at about 11.95 min, but an extra peak still existed at a retention time of about 19.9 min. Both peaks did not appear on the samples from dialysis day 7. These results suggest that the denatured GMA for the same method with synthesized silk fibroin and GMA creates the extra peak in the presence of the original peak with standard GMA. Additionally, both peaks of the GC-MS chromatogram were not detected according to dialysis periods. 

For the MS characterization of these two peaks, the same abundance patterns appeared as for the results from the GC chromatogram. In detail, these differences between two types of standards concerning mass spectra results showed the same *m*/*z* value of 69 *m*/*z* (Table 2). On dialysis days 0, 1, and 2, the samples exhibited the same value of 69 *m*/*z* on the two peaks in the GC results. The retention time at 69 *m*/*z* of the two peaks was 11.99 min and 19.76 min on dialysis day 0, and 11.95 min and 19.83 min on dialysis day 1. On dialysis days 2 and 4, the original peaks disappeared; however, extra peaks still existed at 19.93 min on dialysis day 2, and 19.95 min on dialysis day 4. These two distinct peaks for MS results were not detected on dialysis day 7 with the same results of the GC chromatogram. These GC-MS chromatogram results showed that residual GMA in a Silk-GMA solution decreased gradually concerning the abundance value depending on dialysis periods. At the end of the dialysis periods on 7 day, no other peaks indicating residual GMA were found. These results from GC-MS analysis indicate that the consequences of dialysis periods could eliminate the residual GMA from modified SF and GMA.

### 3.3. Cell Proliferation and Viability of NIH 3T3 Cells with Dialysis Periods of Silk-GMA

**Figure 6:**
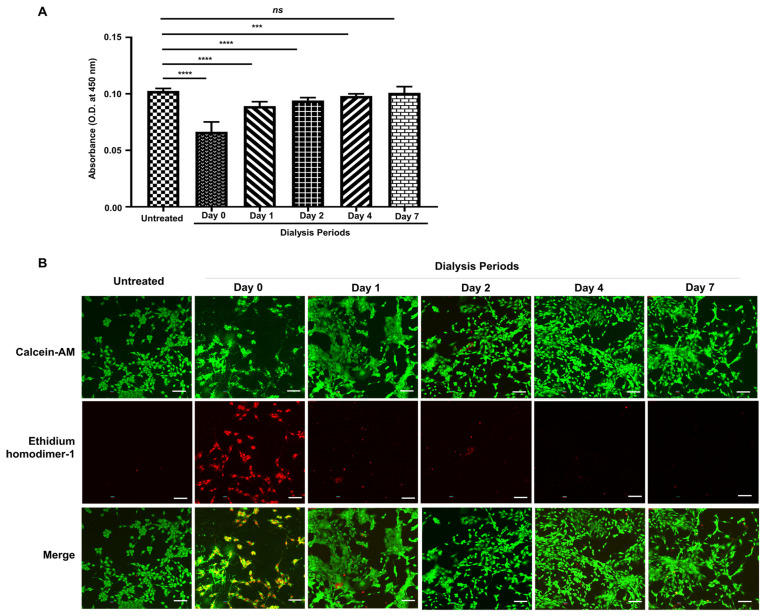
Cell proliferation and viability assay with NIH 3T3 on each dialysis period of modified silk fibroin with glycidyl methacrylate (Silk-GMA). (**A**). CCK 8 assay for cell proliferation rate gradually increased following dialysis period days 0, 1, 2, 4, and 7. **** *p* < 0.0001, *** *p* < 0.001, and ns = no significant. (**B**). NIH 3T3 cells were exposed with 0.1 g/mL of each dialyzed Silk-GMA sample for 72 h. NIH 3T3 cells were detected with Live & Dead assay with indication of calcein AM (live cells, green fluorescent protein) and ethidium homodimer-1 (dead cells, red fluorescent protein), separately. The confocal microscopic images showed that dead cells decreased, and then live cells proliferated gradually depending on dialysis periods (scale bar = 100 µm).

To determine the cell proliferation and viability with each dialyzed sample for Silk-GMA, NIH 3T3 cells were exposed with 0.1 g/mL of each dialyzed Silk-GMA sample for 72 h (Figure 6). Figure 6A presents the proliferation of each dialysis period with Silk-GMA. The proliferation activity of each sample from dialysis periods was 0.1025 ± 0.00226 on untreated samples as a control, 0.06644 ± 0.00876 on dialysis day 0, 0.08915 ± 0.00391 on dialysis day 1, 0.09422 ± 0.00245 on dialysis day 2, 0.09814 ± 0.00189 on dialysis day 4, and 0.1009 ± 0.00553 on dialysis day 7. The proliferation activity of the NIH 3T3 cells with various dialyzed solutions gradually increased according to dialysis periods. Moreover, for the confirmation of cell viability with each dialysis sample from Silk-GMA, we performed Live & Dead assay using confocal microscopic observation (Figure 6B). Live cells in green fluorescence (Calcein AM) and dead cells in red fluorescence (Ethidium homodimer-1) showed significant differences among dialysis days 0 to 7. NIH 3T3 cells on dialysis day 0 presented lots of dead cells in red fluorescence; however, live cells in green fluorescence gradually increased from dialysis day 2. These results showed that the cell proliferation rate increased due to the elimination of residual unreacted chemicals by dialysis period.

### 3.4. Apoptotic and Necrotic Detection with Flow Cytometric Analysis 

**Figure 7:**
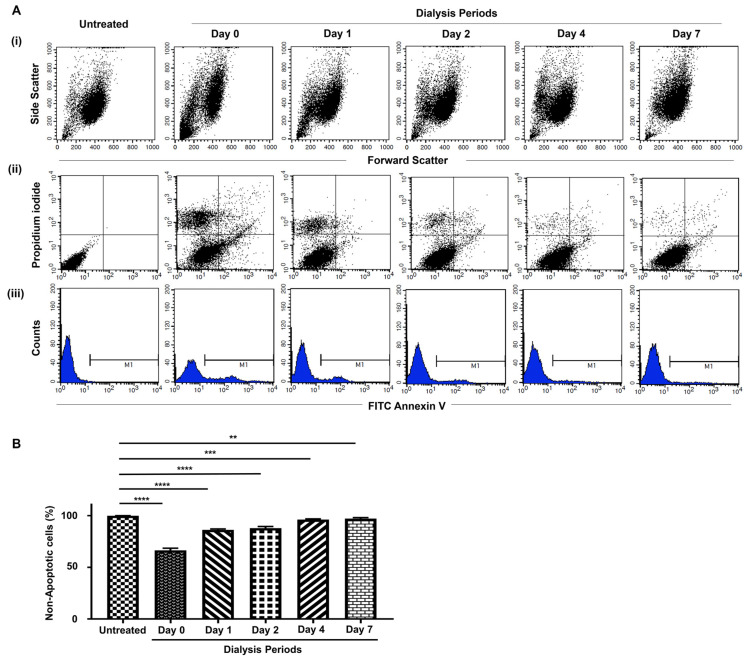
Flow cytometric analysis of necrotic and apoptotic population with Annexin V/propidium iodide staining depending on dialysis periods. (**A**). NIH 3T3 cells treated with 0.1 g/mL of each dialyzed sample on days 0, 1, 2, 4, and 7 for 72 h. (i) The necrotic and apoptotic cell population showed on the light scattering plot; (ii) and (iii) the intensity and percentage of live cells populations (Annexin V-positive) increased gradually following dialysis periods. (**B**). The percentage of necrotic and apoptotic cells were high on dialysis day 0 compared to the untreated sample, and gradually decreased following dialysis periods. **** *p* < 0.0001, *** *p* < 0.001, and ** *p* < 0.05, respectively.

For further investigation, NIH 3T3 cells were treated with 0.1 g/mL of the samples from dialysis days 0, 1, 2, 4, and 7, involving Silk-GMA for 72 h. Then flow cytometric analysis was performed with FITC-annexin V and propidium iodide (PI) double staining (Figure 7). The light-angle scattering plot graph shows (Figure 7Ai) that the population of NIH 3T3 cells gradually increased in forward-angle light scatter (FSC) but decreased in side-angle light scatter (SSC). Figure 7Aii indicates (i) the lower left (FITC-annexin V −/PI −) for viable cells, (ii) the lower right (FITC-annexin V +/PI −) for early apoptotic cells, (iii) the upper right (FITC-annexin V +/PI +) for necrotic or late apoptotic cells, and (iv) the upper left (FITC-annexin V −/PI +) for necrotic cells [27]. The necrotic and apoptotic cell population on dialysis day 0 showed high expression on the fluorescent intensity with FITC-annexin V/PI. Unlike the dialysis day 0 population, the necrotic and apoptotic cell populations of the samples from dialysis days 1 to 7 showed a gradual decrease in the expression of FITC-annexin V/PI from the fluorescent intensity plot graph (Figure 7Aii). These same results were found with the histogram—the percentage of necrotic and apoptotic cells gradually decreased (Figure 7Aiii). The statistical results of the live cell populations were 66.45 ± 2.075% on the samples from dialysis day 0, 86.34 ± 0.8815% on dialysis day 1, 87.972 ± 1.574% on dialysis day 2, 96.34 ± 0.6354% on dialysis day 4, and 97.15 ± 0.9707% on dialysis day 7, compared to the untreated group, 99.96 ± 0.0505%, respectively. These results show that relevant dialysis periods along with the removal of unreacted GMA result in a decrease of cellular apoptosis and necrosis. 

Silk fibroin (SF) from *Bombyx mori* is a natural polymer composed of two proteins, sericin and fibroin. Due to the biocompatibility, high mechanical properties, biodegradable properties, excellent biological characteristics, such as proliferation of various cells, and low inflammation effect, SF has been used in biomedical and tissue engineering. Additionally, SF has been modified with various methods using physical and chemical modifications for the enhancement of mechanical properties as much as native tissue for hydrogel as a biomaterial. In particular, SF can be easily processed into various forms, structures of amino acid modification, and 3D printing applications with the addition of methacrylate groups to amine groups of SF under aqueous processing [28]. SF has a microstructure composed of hydrophobic and hydrophilic regions. These hydrophilic regions contribute water solubility, elasticity, and toughness of SF; it is unlikely that hydrophobic regions form intermolecular interactions, leading to a conformational transition from random-coil or helix to a β-sheet motif. SF can be defined for distinct conformations, α-helix and random-coil conformations (silk I), and the β-sheet conformation (silk II). Silk I is a metastable form that is water soluble, and silk II is insoluble in water with most stable structures. SF chains are connected to silk II by hydrogen bonds between the polypeptide chains [29]. The β-sheet motif of SF functions as a physical cross-link by interconnecting between the SF molecules into a 3D network, providing the high mechanical strength and toughness of SF hydrogels [30]. 

For the extraction of SF, concentrated acid, aqueous, organic, and aqueous–organic salt solutions have been used due to insolubility in water and in the majority of organic solvents [31]. A strong salt solution (LiBr, LiSCN, and HFIP) and calcium nitrate-methanol can be used to make aqueous SF solutions following the degumming procedure. The degumming process is the thermo–chemical process with a strong salt solution under high temperature such as boiling. This process eliminates sericin and other impurities from the silk fibres [32]. Moreover, chemically modified SF hydrogel affects the damages to the encapsulated cells or the surrounding tissues after implantation due to the unreacted chemical residues and released chemical debris during the chemical modifications with a cross-linker. These unreacted strong salt solutions and cross-linkers affect the SF conformational transition [33]. The dialysis process is a key element of SF gelation and is important for providing the mechanical properties of hydrogels. After the dialysis of SF solution with GMA, Silk I, which conformed α-helix and random-coil could be predominant and also easily change to a conformational transition of Silk II, concerning β-sheets with a more stable formation [34].

Concerning further application of Silk-GMA by 3D bioprinting, amino acids with reactive groups that can be modified by GMA are tyrosine, aspartic acid, glutamic acid, threonine, serine, lysine, and arginine. The reactive side chains, such as amine (-NH2), hydroxy (-OH), and carboxyl (-COOH) groups of SF were modified with GMA via an epoxide ring opening reaction. The modification of SF and GMA with the reaction of amine side groups and methacrylate groups can be applied for the 3D bioprinting with the crosslinking site for the photopolymerization. Vinyl groups of GMA are photoreactive groups for UV crosslinking. These vinyl groups of GMA bind to the amine groups on SF after opening of the epoxide ring, resulting in secondary or tertiary amines with hydroxyl groups. Using a photoinitiator, such as LAP (Lithium phenyl(2,4,6-trimethylbenzoyl) phosphinate), the vinyl double bonds on GMA react with each other intra-chain or between chains. SF chains themselves could form the intra-chain connection via vinyl double bonds on GMA reacting during photopolymerization [16]. A natural polymer with chemical modification or dealing with strong acidic or basic solvents for the liquefied polymer is needed to perform the evaluation of biological safety.

## 4. Conclusions

Silk fibroin has been widely used s a biomedical and bio-industrial application because the degummed SF has strong advantages such as biocompatibility, biodegradability, mechanical strength, and biological characteristics. Moreover, SF could be modified into various forms with chemical modification. In this study, we evaluated the effect of the residual chemical cross-linker on the changing of characteristics, residual detection, and cytocompatibilities including cell proliferation and cytotoxicity by detection of necrotic and apoptotic cells of modified silk fibroin with glycidyl methacrylate. We also investigated the importance of dialysis of a natural polymer with chemical modification to increase cellular activity and ensure safety for the trial. Dialysis periods from days 2, 4, and 7 showed defined relevant signals for FT−IR results; functional amine groups were identified. Through the dialysis periods, the elimination of residual unreacted GMA has been confirmed. Increments in the cell proliferation rate and decreases in necrotic and apoptotic cells depended on dialysis periods. Therefore, we suggest that the ideal dialysis period for the modification of SF and GMA is seven days with a good cell proliferation rate, in accordance with the results from the observation of residual unreacted GMA on gas chromatography–mass spectra. These results indicated that the dialysis procedure and periods affect the cellular biological changes of a scaffold with chemical modification without changing the structural conformation. Additionally, these results suggested the potential application of standardization for chemically modified silk fibroin as a scaffold for the clinical trials for tissue engineering and biomedical applications.

## Figures and Tables

**Table 1:** Gas chromatography and mass spectra (GC-MS) conditions.

**Gas Chromatograph**	Instruments	7820A	
	Column	Type	Fused silica HP-5ms GC column
		Length	30 m
		Inner Diameter (mm)	0.25 mm
		Film Thickness (µm)	0.25 µm
	Carrier gas	Gas type	Helium (99.999%)
		Flow rate	1.0 mL/min
	Inlet	Temperature	280 °C
		Injection volume	1 µL
		Injection mode	Split
		Split ratio	10:1
	Temperature program	Temperature gradient	60 °C for 5 min; increased up to 200 °C for 5 min with 5 °C per min rate
		Final heating	230 °C
	Analysis software	ChemStation	
**Mass Spectra**	Instruments	MSD 5977E	
	Ionization mode	Electron Impact (EI)	
	Electron energy	70 eV	
	Analytical mode	SCAN	
	Source Temperature	230 °C	
	Solvent delay time	3.0 min	
	Scanning range (m/z)	50 to 500 mass to charge ratio (m/z)	
	Analysis software	Mass Hunter	

**Table 2:** The GC-MS analysis of retention time and abundance value for dialysis days 0, 1, 2, 4, and 7. Analyses were compared with GMA and denatured GMA standard at 60 °C for 6 h, and each dialyzed sample of Silk-GMA. GMA, glycidyl methacrylate; ND, not detected.

Standard		Gas Chromatograph				Mass Spectra	
		Retention Time (min)		Abundance (×10^6^)		Abundance (×10^6^)	
		1st Peak	2nd Peak	1st Peak	2nd Peak	1st Peak	2nd Peak
GMA Standard		11.95 ± 0.144	ND	6.5 ± 0.013	ND	5.5 ± 0.017	ND
Denatured GMA Standard		11.973 ± 0.136	19.931 ± 0.126	0.5 ± 0.024	0.15 ± 0.012	0.28 ± 0.011	0.06 ± 0.015
Dialysis	Day 0	11.997 ± 0.095	19.763 ± 0.104	0.09 ± 0.011	0.12 ± 0.009	0.036 ± 0.03	0.055 ± 0.017
	Day 1	11.949 ± 0.125	19.831 ± 0.161	0.019 ± 0.016	0.049 ± 0.0101	0.011 ± 0.01	0.032 ± 0.010
	Day 2	ND	19.937 ± 0.144	ND	0.025 ± 0.004	ND	0.014 ± 0.023
	Day 4	ND	19.955 ± 0.099	ND	0.011 ± 0.006	ND	0.0095 ± 0.007
	Day 7	ND	ND	ND	ND	ND	ND

## Data Availability

The authors declare that all data of this study are available within the article from the corresponding author on reasonable request.
